# An *in silico* evaluation of the ADMET profile of the StreptomeDB database

**DOI:** 10.1186/2193-1801-2-353

**Published:** 2013-07-30

**Authors:** Fidele Ntie-Kang

**Affiliations:** Chemical and Bioactivity Information Centre, Department of Chemistry, Faculty of Science, University of Buea, P. O. Box 63, Buea, Cameroon; Department of Pharmaceutical Sciences, Martin-Luther University of Halle-Wittenberg, Wolfgang-Langenbeck Str. 4, 06120 Halle (Saale), Germany; CEPAMOQ, Faculty of Science, University of Douala, P.O. Box 8580, Douala, Cameroon

**Keywords:** 3D structures; *Streptomyces*, Database, *In silico*, Natural products, Virtual screening

## Abstract

**Background:**

Computer-aided drug design (CADD) often involves virtual screening (VS) of large compound datasets and the availability of such is vital for drug discovery protocols. This paper presents an assessment of the “drug-likeness” and pharmacokinetic profile of > 2,400 compounds of natural origin, currently available in the recently published StreptomeDB database.

**Methods:**

The evaluation of “drug-likeness” was performed on the basis of Lipinski’s “Rule of Five”, while 46 computed physicochemical properties or molecular descriptors were used to predict the absorption, distribution, metabolism, elimination and toxicity (ADMET) of the compounds.

**Results:**

This survey demonstrated that, of the computed molecular descriptors, about 28% of the compounds within the StreptomeDB database were compliant, having properties which fell within the range of ADMET properties of 95% of currently known drugs, while about 44% of the compounds had ≤ 2 violations. Moreover, about 50% of the compounds within the corresponding “drug-like” subset showed compliance, while >83% of the “drug-like” compounds had ≤ 2 violations.

**Conclusions:**

In addition to the previously verified range of measured biological activities, the compounds in the StreptomeDB database show interesting DMPK profiles and hence could represent an important starting point for hit/lead discovery from natural sources. The generated data are available and could be highly useful for natural product lead generation programs.

**Electronic supplementary material:**

The online version of this article (doi:10.1186/2193-1801-2-353) contains supplementary material, which is available to authorized users.

## Background

Many drugs often fail to enter the market as a result of poor pharmacokinetic profiles (Darvas et al. 2002). Thus, it has become imperative nowadays to design lead compounds which can be easily orally absorbed, easily transported to their desired site of action, not easily metabolised into toxic metabolic products before reaching the targeted site of action and easily eliminated from the body before accumulating in sufficient amounts that may produce adverse side effects. The sum of the above mentioned properties is often referred to as ADME (absorption, distribution, metabolism and elimination) properties, or better still ADMET, ADME/T or ADMETox (when considerations are given to toxicity issues). The inclusion of pharmacokinetic considerations at earlier stages of drug discovery programs (Hodgson 2001; Navia and Chaturvedi 1996) using computer-based methods is becoming increasingly popular (Lipinski et al. 1997; Lombardo et al. 2003; Gleeson et al. 2011). The rationale behind *in silico* approaches are the relatively lower cost and the time factor involved, when compared to standard experimental approaches for ADMET profiling (DiMasi et al. 2003; Darvas et al. 2002). As an example, it only takes a minute in an *in silico* model to screen 20,000 molecules, but takes 20 weeks in the “wet” laboratory to do the same exercise (Hodgson 2001).

Due to the accumulated ADMET data in the late 1990s, many pharmaceutical companies are now using computational models that, in some cases, are replacing the “wet” screens (Hodgson 2001). This paradigm shift has therefore spurred up the development of several theoretical methods for the prediction of ADMET parameters. A host of these theoretical models have been implemented in a number of software programs currently available for drug discovery protocols ( OCHEM platform; Lhasa 2010; Schrödinger 2011a; Cruciani et al. 2000), even though some of the predictions are often disappointing (Tetko et al. 2006). The software tools currently used to predict the ADMET properties of potential drug candidates often make use of quantitative structure-activity relationships, QSAR (Tetko et al. 2006; Hansch et al. 2004) or knowledge-base methods (Greene et al. 1999; Button et al. 2003; Cronin 2003). A promising lead compound may therefore be defined as one which combines potency with an attractive ADMET profile. As such, compounds with uninteresting predicted ADMET profiles may be completely dismissed from the list of potential drug candidates early enough (even if these prove to be highly potent). Otherwise, the DMPK properties are “fine-tuned” in order to improve their chances of making it to clinical trials (Hou and Wang 2008).

In this paper, we assess the pharmacokinetic profile of the recently published StreptomeDB database (Lucas et al. 2013) using an *in silico* method. A number of computed molecular descriptors, currently implemented in a wide range of software, have been used as indicators of the pharmacokinetic properties of a large proportion of currently known drugs.

## Methods

### Data source and initial treatment of chemical structures

The 2,444 3D structures of the compounds in the StreptomeDB database were downloaded from the official webpage of the Pharmaceutical Bioinformatics group of the University of Freiburg (http://www.pharmaceutical-bioinformatics.de/streptomedb/). These were initially treated with LigPrep (Schrödinger 2011b). The implementation was carried out with the graphical user interface (GUI) of the Maestro software package (Schrödinger 2011c), using the OPLS forcefield (Shivakumar et al. 2010; Jorgensen et al. 1996; Jorgensen and Tirado-Rives 1988). Protonation states at biologically relevant pH were correctly assigned (group I metals in simple salts were disconnected, strong acids were deprotonated, strong bases protonated, while topological duplicates and explicit hydrogens were added). All molecular modelling was carried out on a Linux workstation with a 3.5 GHz Intel Core2 Duo processor.

### Calculation of ADMET-related descriptors

A set of ADMET-related properties (a total of 46 molecular descriptors) were calculated by using the QikProp program (Schrödinger 2011d) running in normal mode. QikProp generates physically relevant descriptors, and uses them to perform ADMET predictions. An overall ADME-compliance score – drug-likeness parameter (indicated by #stars), was used to assess the pharmacokinetic profiles of the compounds within the StreptomeDB library. The #stars parameter indicates the number of property descriptors computed by QikProp that fall outside the optimum range of values for 95% of known drugs. The methods implemented were developed by Jorgensen and Duffy (Jorgensen and Duffy 2002; Duffy and Jorgensen 2000; Jorgensen and Duffy 2000) and among the calculated descriptors are: the total solvent-accessible molecular surface, S_*mol*_ in Å^2^ (probe radius 1.4 Å) (range for 95% of drugs: 300–1000 Å^2^); the hydrophobic portion of the solvent-accessible molecular surface, S_*mol*,*hfob*_ in Å^2^ (probe radius 1.4 Å) (range for 95% of drugs: 0–750 Å^2^); the total volume of molecule enclosed by solvent-accessible molecular surface, V_*mol*_ in Å^3^ (probe radius 1.4 Å) (range for 95% of drugs: 500–2000 Å^3^); the logarithm of aqueous solubility, log S_*wat*_ (range for 95% of drugs: -6.0 to 0.5) (Jorgensen and Duffy 2002; Jorgensen and Duffy 2000); the logarithm of predicted binding constant to human serum albumin, log *K*_*HSA*_ (range for 95% of drugs: -1.5 to 1.2) (Colmenarejo et al. 2001); the logarithm of predicted blood/brain barrier partition coefficient, log B/B (range for 95% of drugs: -3.0 to 1.0) (Luco 1999; Kelder et al. 1999; Ajay et al. 1999); the predicted apparent Caco-2 cell membrane permeability (BIP_*caco* − 2_) in Boehringer–Ingelheim scale, in nm s^-1^ (range for 95% of drugs: < 5 low, > 100 high) (Yazdanian et al. 1998; Irvine et al. 1999; Stenberg et al. 2001); the predicted apparent Madin-Darby canine kidney (MDCK) cell permeability in nm s^-1^ (< 25 poor, > 500 great) (Irvine et al. 1999); the index of cohesion interaction in solids, Ind_coh_, calculated from the number of hydrogen bond acceptors (HBA), donors (HBD) and the surface area accessible to the solvent, SASA (*S*_*mol*_) by the relation  (0.00 to 0.05 for 95% of drugs) (Jorgensen and Duffy 2000); the globularity descriptor, Glob = (4*πr*^2^)/*S*_*mol*_, where *r* is the radius of the sphere whose volume is equal to the molecular volume (0.75 to 0.95 for 95% of drugs); the predicted polarizability, *QP*_*polrz*_ (13.0 to 70.0 for 95% of drugs); the predicted IC_50_ value for blockage of HERG K^+^ channels, log *HERG* (concern < −5) (Cavalli et al. 2002; De Ponti et al. 2001); the predicted skin permeability, log *K*_p_ (−8.0 to −1.0 for 95% of drugs) (Potts and Guy 1992; Potts and Guy 1995); and the number of likely metabolic reactions, #metab (range for 95% of drugs: 1–8).

## Results and discussion

### Drug-likeness assessment

**Figure 1:**
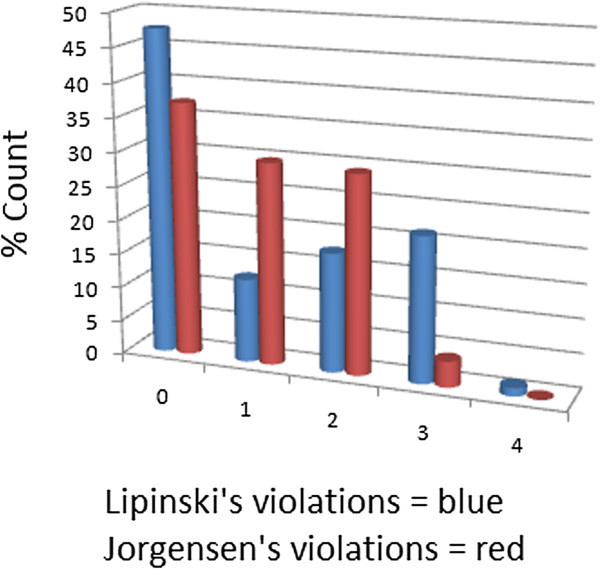
**Distributions of violations of Lipinski’s ro5 and Jorgensen’s ro3 within the StreptomeDB database.**

**Figure 2:**
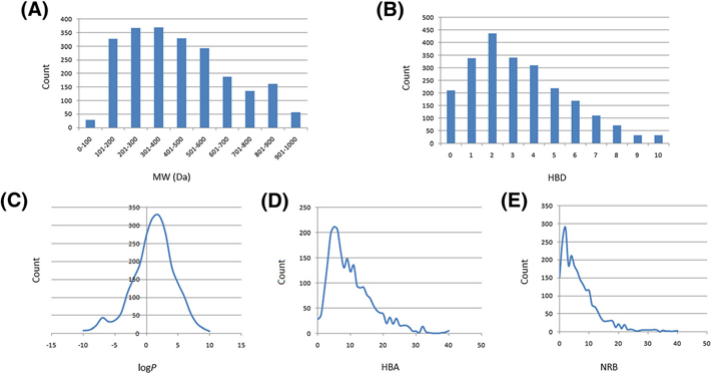
**Distributions of features that determine “drug-likeness” in StreptomeDB. (A)** MW, **(B)** HBD, **(C)** log *P*, **(D)** HBA, **(E)** NRB.

**Figure 3:**
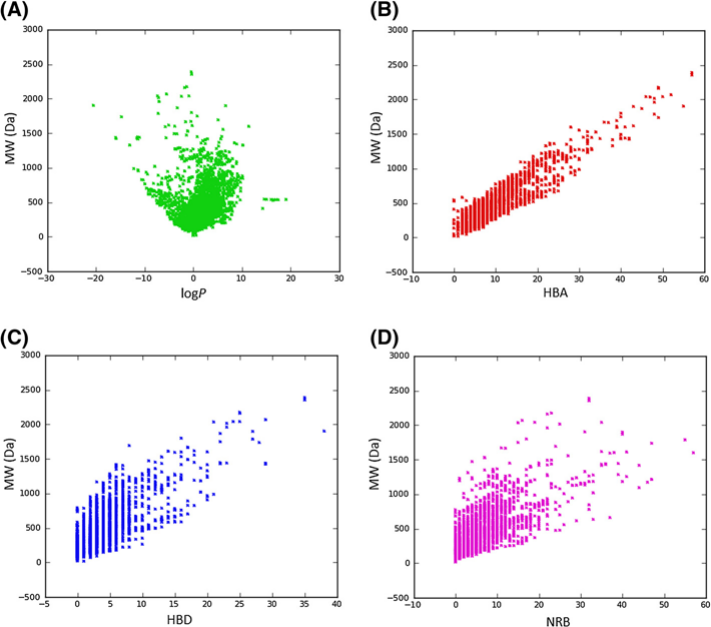
**Scatter diagrams showing pair wise distribution of “drug-likeness” descriptors. (A)** MW against log *P*, **(B)** MW against HBA, **(C)** MW against HBD and **(D)** MW against NRB.

The “drug-likeness” test was carried out using Lipinski’s “Rule of Five”, ro5 (Lipinski et al. 1997). The distributions of the compound molecular weights (MW), calculated lipophilicity (log *P*), number of hydrogen bond acceptors (HBA) and number of hydrogen bond donors (HBD) were used to assess the “drug-likeness” of StreptomeDB. It is noteworthy that natural products exhibit a wide range of flexibility, from rigid conformationally constrained molecules to very flexible compounds. Thus, the number of rotatable bonds (NRB) within the StreptomeDB library was used as an additional criterion to test for the favourable drug metabolism and pharmacokinetics (DMPK) outcomes. It was observed that 47.5% of the compounds within StreptomeDB showed no Lipinski violations and 77.3% showed ≤ 2 violations (Figure 1), while the peak of the distribution of the NRB was between 1 and 2 (Figure 2E). Moreover, the analysis of the distributions of MW (truncated at MW = 1000 Da for the sake of clarity), showed a peak value between 301 and 400 Da (Figure 2A), with a curve similar to those previously reported for other “drug-like” NP libraries in the literature (Ntie-Kang et al. 2013; Quinn et al. 2008; Feher and Schmidt 2003) and about 42% of MW > 500 Da. The distribution of the log *P* values showed a Gaussian shaped curve with a peak centred at 2.5 log *P* units (Figure 2C). However, some of the compounds had exceptionally large log *P* values (truncated at 10 log *P* units for the sake of clarity), which went up to > 19 units. This may be explained by the fact that the training database/algorithm used to calculate log *P* may not suit the types and combinations of functional groups found in natural products (Quinn et al. 2008). It should however be noted that, inspite of this limitation, 85.3% of the compounds from StreptomeDB had log *P* values < 5 units. The peaks of the HBA and HBD were respectively at 5 acceptors and 2 donors and both curves fell off rapidly to maximum numbers of 57 and 38 respectively (truncated at 40 HBA and 10 HBD respectively, Figure 2B and 2D). It was also noted that ~40% of the compounds in StreptomeDB had HBA > 10 and only ~24% had HBD > 5. Additionally, the pairwise comparison displaying the mutual relationship between the molecular weight versus the calculated log *P*, HBA, HBD and NRB are specified in Figure 3A-D, respectively. The plots show that the regions with the highest population densities fall within the “Lipinski region of interest” (MW < 500, -2 < log P < 5, HBA < 10 and HBD < 5), and for which NRB < 5.

### Overall DMPK compliance of the SreptomeDB library

**Figure 4:**
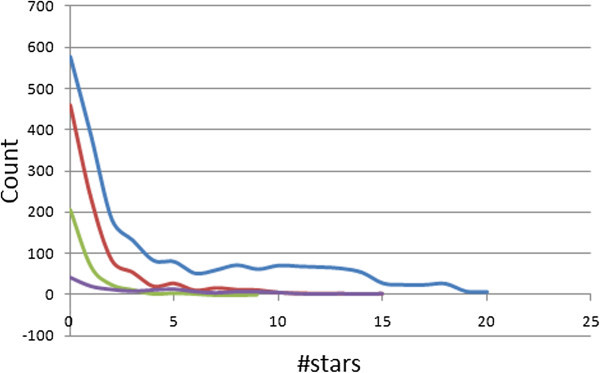
**Distribution curves for #stars within the StreptomeDB library, along with the standard “drug-like”, “lead-like” and “fragment-like” subsets.** Blue = StreptomeDB library, red = “drug-like” subset, green = “lead-like” subset and violet = “fragment-like” subset.

The 24 most relevant molecular descriptors calculated by QikProp are used to determine the #star parameter (Schrödinger 2011d). A plot of the #stars parameter (on *x*-axis) against the corresponding counts (on *y*-axis) in the StreptomeDB is plotted within the same set of axes with those of the “drug-like”, “lead-like”, and “fragment-like” standard subsets, as shown in Figure 4. The criteria for the respective standard subsets were defined as (MW < 500; log *P* < 5; HBD ≤ 5; HBA ≤ 10) (Lipinski et al. 1997), (150 ≤ MW ≤ 350; log *P* ≤ 4; HBD ≤ 3; HBA ≤ 6) (Teague et al. 1999; Oprea 2002; Schneider 2002) and (MW ≤ 250; -2 ≤ log *P* ≤ 3; HBD < 3; HBA < 6; NRB < 3) (Verdonk et al. 2003). The ADMET descriptors for some 316 compounds in the total library were not computed by QikProp. This could be due to technical difficulties with running the software, due to errors in the input structures downloaded from the StreptomeDB website. Of the remaining 2,128 compounds, 27.2% showed #star = 0, while 53.8% had #star ≤ 2. Among the 925 compounds of the “drug-like” subset whose pharmacokinetic properties were predicted, 49.7% had pharmacokinetic descriptors within the acceptable range for 95% of known drugs, while 83.5% showed #stars ≤ 2. The “lead-like” and “fragment-like” subsets were respectively 63.5% and 31.5% compliant for all of the 24 most relevant computed descriptors. The average values for 19 selected computed descriptors have been shown in Table 1 for all 4 compound libraries. The average values indicate a high probability of finding drug leads within the StreptomeDB compound library.

**Table 1:** **Summary of average pharmacokinetic property distributions of the total StreptomeDB library in comparison with the various subsets**

Library name	^***a***^Lib. size	^***b***^No. compl.	^***c***^MW (Da)	^***d***^Log***P***	^***e***^HBA	^***f***^HBD	^***g***^NRB
**StreptomeDB**	2,444	577	485	1.30	11.69	3.47	11.46
**Drug-like**	925	459	262	1.19	5.27	1.92	4.78
**Lead-like**	326	207	230	1.61	3.96	1.46	3.68
**Fragment-like**	127	40	151	0.96	2.99	1.07	1.28
**Library name**	^***h***^**LogB/B**	^***i***^**BIP**_**caco**-**2**_**(nm s**^-**1**^)	^***j***^***S***_**mol**_**(Å**^**2**^)	^***k***^***S***_**mol,hfob**_**(Å**^**2**^)	^***l***^***V***_**mol**_**(Å**^**3**^)	^***m***^**Log*S***_***wat***_**(S in mol L**^-**1**^)	^***n***^**Log*K***_***HSA***_
**StreptomeDB**	−2.25	522	748	417	1426	−3.20	−0.48
**Drug**-**like**	−1.01	734	490	213	840	−2.42	−0.37
**Lead**-**like**	−0.72	986	460	179	768	−2.50	−0.29
**Fragment**-**like**	−0.31	1275	343	112	536	−1.14	−0.63
**Library name**	^**o**^**MDCK**	^***p***^**Ind**_**coh**_	^***q***^**Glob**	^***r***^**QP**_**polrz**_**(Å**^**3**^)	^***s***^**LogHERG**	^***t***^**Log*K***_***p***_	^***u***^**#** **metab**
**StreptomeDB**	368	0.028	0.82	44.53	−3.90	−4.76	6.62
**Drug**-**like**	545	0.015	0.88	25.44	−3.33	−3.93	3.48
**Lead**-**like**	638	0.010	0.88	23.56	−3.59	−3.28	2.75
**Fragment**-**like**	896	0.008	0.93	15.59	−2.45	−2.98	1.45

^*a*^Size or number of compounds in library; ^*b*^Number of compounds with #star = 0; ^*c*^Molar weight (range for 95% of drugs: 130–725 Da); ^*d*^Logarithm of partitioning coefficient between *n*-octanol and water phases (range for 95% of drugs: -2 to 6); ^*e*^Number of hydrogen bonds accepted by the molecule (range for 95% of drugs: 2–20); ^*f*^Number of hydrogen bonds donated by the molecule (range for 95% of drugs: 0–6).; ^*g*^Number of rotatable bonds (range for 95% of drugs: 0–15); ^*h*^Logarithm of predicted blood/brain barrier partition coefficient (range for 95% of drugs: -3.0 to 1.0); ^*i*^Predicted apparent Caco-2 cell membrane permeability in Boehringer–Ingelheim scale, in nm/s (range for 95% of drugs: < 5 low, > 100 high); ^*j*^Total solvent-accessible molecular surface, in Å^2^ (probe radius 1.4 Å) (range for 95% of drugs: 300–1000 Å^2^); ^*k*^Hydrophobic portion of the solvent-accessible molecular surface, in Å^2^ (probe radius 1.4 Å) (range for 95% of drugs: 0–750 (Å^2^); ^*l*^Total volume of molecule enclosed by solvent-accessible molecular surface, in Å^3^ (probe radius 1.4 Å) (range for 95% of drugs: 500–2000 Å^3^); ^*m*^Logarithm of aqueous solubility (range for 95% of drugs: -6.0 to 0.5); ^*n*^Logarithm of predicted binding constant to human serum albumin (range for 95% of drugs: -1.5 to 1.2); ^*o*^Predicted apparent MDCK cell permeability in nm/sec (< 25 poor, > 500 great); ^*p*^Index of cohesion interaction in solids (0.0 to 0.05 for 95% of drugs); ^*q*^Globularity descriptor (0.75 to 0.95 for 95% of drugs); ^*r*^Predicted polarizability (13.0 to 70.0 for 95% of drugs); ^*s*^Predicted IC_50_ value for blockage of HERG K^+^ channels (concern < −5); ^*t*^Predicted skin permeability (−8.0 to −1.0 for 95% of drugs); ^*u*^Number of likely metabolic reactions (range for 95% of drugs: 1–8).

### Bioavailability prediction

**Figure 5:**
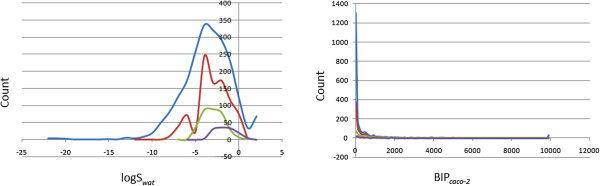
**Distribution curves for compliance to Jorgensen’s “Rule of Three” (A) calculated log S**_**wat**_**against count, (B) predicted BIP**_**caco − 2**_**against count.** Colour codes are as defined in Figure 4.

**Figure 6:**
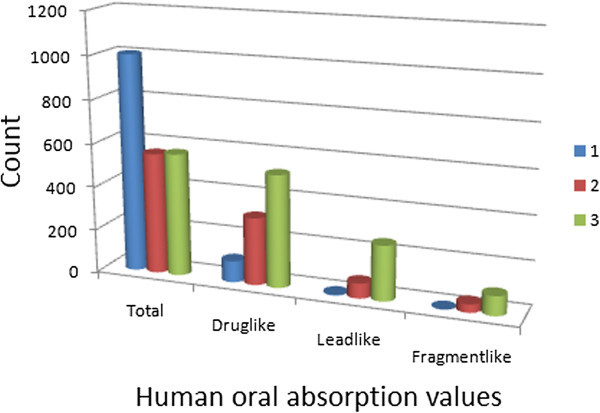
**Histograms showing the distribution of human oral absorption predictions.**

According to Jorgensen’s ro3, if a compound complies to all or some of the rules (log S_*wat*_ > −5.7, BIP_*caco* − 2_ > 22 nm/s and # Primary Metabolites < 7), then it is more likely to be orally available (Jorgensen and Duffy 2000; Jorgensen and Duffy 2002; Schrödinger 2011d). The bioavailability of a compound depends on the processes of absorption and liver first-pass metabolism (Van de Waterbeemd and Gifford 2003). Absorption in turn depends on the solubility and permeability of the compound, as well as interactions with transporters and metabolizing enzymes in the gut wall. The computed parameters used to assess oral absorption are the predicted aqueous solubility, log S_*wat*_, the conformation-independent predicted aqueous solubility, CI log S_*wat*_, the predicted qualitative human oral absorption, the predicted % human oral absorption and compliance to Jorgensen’s famous “Rule of Three” (ro3). The solubility calculation procedure implemented depends on the similarity property space between the given molecule and its most similar analogue within the experimental training set used to develop the model implemented in QikProp, i.e., if the similarity is < 0.9, then the QikProp predicted value is taken, otherwise, the predicted property, *P*_*pred*_ , is adjusted such that:1

where *S* is the similarity, and *P*_*exp*_ and *P*_*QP*_ are the respective experimental and QikProp predictions for the most similar molecule within the training set. In equation (1), if *S* = 1, then the predicted property is equal to the measured experimental property of the training set compound. The distribution curves for two of the three determinants for the ro3 (log S_*wat*_ and BIP_*caco* − 2_) are shown in Figure 5. In general 37.3% of the StreptomeDB library was compliant to the ro3, while the respective % compliances for the various subsets were 72.7%, 92.3% and 97.6% for the “drug-like”, “lead-like” and “fragment-like” libraries. Among the individual computed parameters, the most remarkable was log S_*wat*_, which was met by 85.1% of the compounds within the StreptomeDB library. This property showed a Gaussian distribution for the “drug-like” and “lead-like” subsets. Only 39.9% of the compounds fell within the respected range for the BIP_*caco* − 2_ criterion. The predicted apparent Caco-2 cell permeability, BIP_*caco* − 2_ (in nm s^-1^), model the permeability of the gut-blood barrier (for non-active transport), even though this parameter is not often correctly predicted computationally (Veber et al. 2002). The histograms of the predicted qualitative human oral absorption parameter (in the scale 1 = low, 2 = medium and 3 = high) are shown in Figure 6. It was observed 26.6% of the compounds in the total StreptomeDB were predicted to have high human oral absorption. The predicted% human oral absorption (on 0 to 100% scale) shows a similar trend, 10.9% of the compounds being predicted to be absorbed at 100% and 15.9% of the compounds predicted to be absorbed at > 90%.

A molecule’s size, as well as its capacity to make hydrogen bonds, its overall lipophilicity and its shape and flexibility are important properties to consider when determining permeability. Molecular flexibility has been seen as a parameter which is dependent on the number of rotatable bonds (NRB), a property which influences bioavailability in rats (Veber et al. 2002). The results for distribution of the NRB for this dataset revealed that the compounds within the StreptomeDB library show some degree of conformational flexibility, the peak value for the NRB being between 1 and 2, while the average values is 11.46 (Table 1). The large gap between the peak and average value could be explained by the presence of very huge NPs within the dataset, containing as many as 72 rotatable single bonds (truncated at 40 RBs in Figure 2E).

### Prediction of blood–brain barrier (BBB) penetration

**Figure 7:**
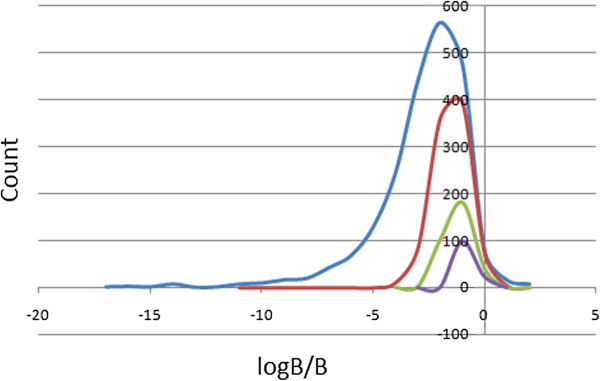
**Plot of the physico-chemical descriptor used to predict BBB penetration.** Predicted log B/B against count. The *x*-axis label is the lower limit of binned data, e.g. 0 is equivalent to 0.0 to 1.0. Colour codes are as defined in Figure 4.

Too polar drugs do not cross the BBB. The blood/brain partition coefficients (log B/B) were computed and used as a predictor for access to the central nervous system (CNS). The predicted CNS activity was computed on a −2 (inactive) to +2 (active) scale and showed that only 2.47% of the compounds in StreptomeDB could be active in the CNS (predicted CNS activity > 1). A distribution of log B/B (Figure 7) shows a right-slanted Gaussian-shaped curve with a peak at −1.5 log B/B units for the total library, and −0.5 log B/B units for the standard subsets, with 73.7% of the compounds in StreptomeDB falling within the recommended range for the predicted brain/blood partition coefficient (−3.0 to 1.2). Madin-Darby canine kidney (MDCK) monolayers, are widely used to make oral absorption estimates, the reason being that these cells also express transporter proteins, but only express very low levels of metabolizing enzymes (Veber et al. 2002). They are also used as an additional criterion to predict BBB penetration. Thus, our calculated apparent MDCK cell permeability could be considered to be a good mimic for the BBB (for non-active transport). It was estimated that only about 35% of the compounds had apparent MDCK cell permeabilities falling within the recommended range of 25–500 nm s^-1^ for 95% of known drugs. This situation knew no improvements in the “drug-like” and “lead-like” subsets.

### Prediction of dermal penetration

**Figure 8:**
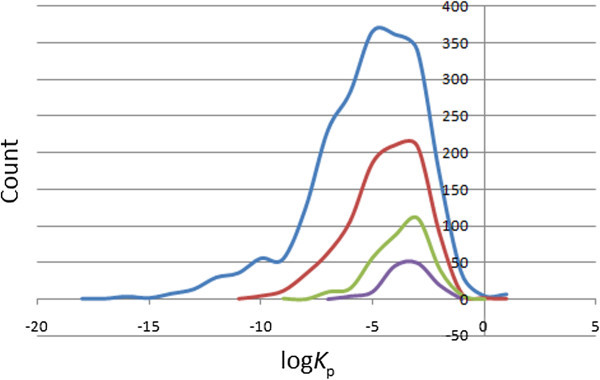
**Distribution curves for the predicted skin penetration parameter.** Colour codes are as defined in Figure 4.

This factor is important for drugs administered through the skin. The distribution of computed skin permeability parameter, log *K*_p_, showed smooth Gaussian-shaped graphs centred at −4.5 log *K*_p_ units for the total database, at −3.5 log *K*_p_ units for the “drug-like” subset and −2.5 log *K*_p_ units for the “lead-like” and “fragment-like” subsets (Figure 8), with ~88% of the compounds in the StreptomeDB database falling within the recommended range for 95% of known drugs. The predicted maximum transdermal transport rates, *J*_*m*_  (in μ cm^-2^ hr^-1^), were computed from the aqueous solubility (S_*wat*_) and skin permeability (*K*_p_), using the relation (2):2

This parameter showed variations from 0 to about 86,100 μ cm^-2^ hr^-1^, with only about 2.7% of the compounds in StreptomeDB having predicted value of *J*_*m*_  > 100 μ cm^-2^ hr^-1^.

### Prediction of plasma-protein binding

**Figure 9:**
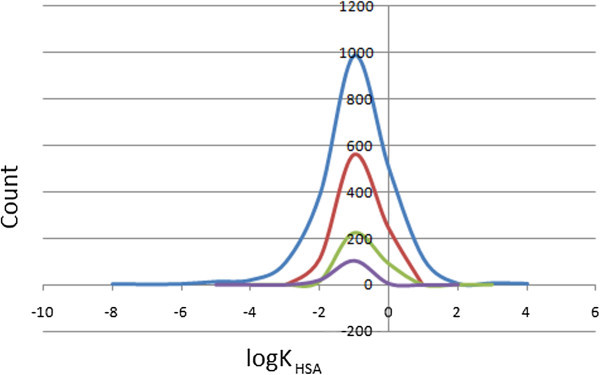
**Distribution curves for predicted plasma-protein binding.** Colour codes are as defined in Figure 4.

The efficiency of a drug may be affected by the degree to which it binds to the proteins within blood plasma. It is noteworthy that binding of drugs to plasma proteins (like human serum albumin, lipoprotein, glycoprotein, α, β, and γ globulins) greatly reduces the quantity of the drug in general blood circulation and hence the less bound a drug is, the more efficiently it can traverse cell membranes or diffuse. The predicted plasma-protein binding has been estimated by the prediction of binding to human serum albumin; the log *K*_HSA_ parameter (recommended range is −1.5 to 1.5 for 95% of known drugs). Figure 9 shows the variation of this calculated parameter within the StreptomeDB dataset, as well as for the standard subsets. This equally gave smooth Gaussian-shaped curves centred on −0.5 log *K*_HSA_ units for all the datasets. In addition, our calculations reveal that > 86% of the compounds within the StreptomeDB library are compliant to this parameter, indicating that a majority of the compounds are likely to circulate freely within the blood stream and hence have access to the target site.

### Metabolism prediction

**Figure 10:**
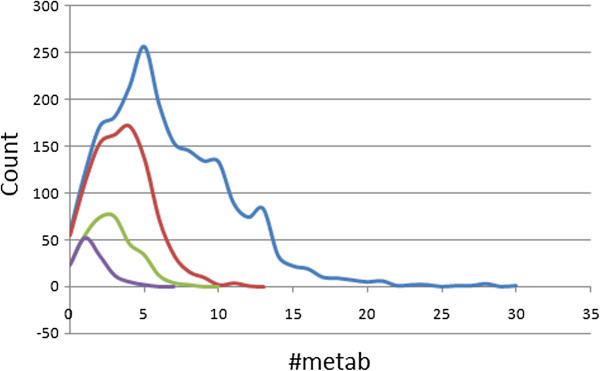
**Graphs showing the distribution of the predicted number of metabolic reactions.** Colour codes are as defined in Figure 4.

An estimated number of possible metabolic reactions has also been predicted by QikProp and used to determine whether the molecules can easily gain access to the target site after entering the blood stream. The average estimated number of possible metabolic reactions for the StreptomeDB library is between 5 and 6, while those of the standard subsets are respectively between 6 and 7, between 3 and 4 and between 1 and 2 for the “drug-like”, “lead-like” and “fragment-like” libraries (Table 1). Even though some of the compounds are likely to undergo as many as up to 30 metabolic reactions due to the complexity of some of the secondary metabolites within the database (Figure 8), ~68% of the compounds are predicted to undergo the recommended number of metabolic steps (1 to 8 reactions), with the situation improving to ~92% and almost 100% in the “drug-like” and “lead-like” subsets respectively. From Figure 10, it can be observed that the total, “drug-like”, “lead-like”, and “fragment-like” libraries both show respective peak values at 5, 4, 3 and 2 metabolic steps.

### Prediction of blockage of human ether-a-go-go-related gene potassium (HERG K^+^) channel

**Figure 11:**
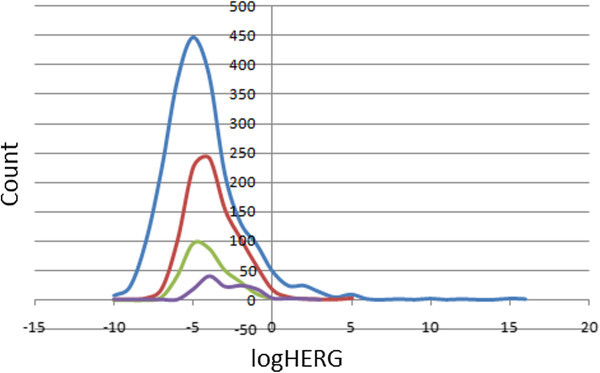
**A plot of predicted logHERG values for StreptomeDB and standard subsets.** Colour codes are as defined in Figure 4.

Human ether-a-go-go related gene (HERG) encodes a potassium ion (K^+^) channel that is implicated in the fatal arrhythmia known as *torsade de pointes* or the long QT syndrome (Hedley et al. 2009). The HERG K^+^ channel, which is best known for its contribution to the electrical activity of the heart that coordinates the heart's beating, appears to be the molecular target responsible for the cardiac toxicity of a wide range of therapeutic drugs (Vandenberg et al. 2001). HERG has also been associated with modulating the functions of some cells of the nervous system and with establishing and maintaining cancer-like features in leukemic cells (Chiesa et al. 1997). Thus, HERG K^+^ channel blockers are potentially toxic and the predicted IC_50_ values often provide reasonable predictions for cardiac toxicity of drugs in the early stages of drug discovery (Aronov 2005). In this work, the estimated or predicted IC_50_ values for blockage of this channel have been used to model the process *in silico*. The recommended range for predicted log IC_50_ values for blockage of HERG K^+^ channels (logHERG) is > −5. A distribution curve for the variation of the predicted logHERG is shown in Figure 11, which is a left-slanted Gaussian-shaped curve, with a peak at −5.5 logHERG units for the total library, as well as for the “drug-like” and “lead-like” subsets. It was observed that in general, this parameter is predicted to fall within the recommended range for about 66% of the compounds within the StreptomeDB dataset, ~87% for the “drug-like” subset and ~86% for the “lead-like” subset.

### Usefulness of the compound library

The treated 3D structures of the compounds, as well as their physico-chemical properties that were used to evaluate the “drug-likeness” and DMPK profile, can be freely downloaded as additional files accompanying this publication (Additional file 1, Additional file 2, Additional file 3, Additional file 4 and Additional file 5). The computed properties included in the attached files could be a useful guide in compound selection during virtual screening campaigns and hence help users carefully select which compounds to further develop in a drug discovery program which begins with the StreptomeDB database.

## Conclusions

Modern drug discovery programs usually involve the search for small molecule leads with attractive pharmacokinetic profiles. The presence of such within the material in the additional files accompanying this publication (for non commercial use) is of major importance and therefore renders the database attractive, in addition to the already known properties – “drug-likeness”, “lead-likeness”, “fragment-likeness” and diversity (Lucas et al. 2013). This is an indication that the 3D structures of naturally occurring compounds within StreptomeDB could be a good starting point for docking, neural networking and pharmacophore-based virtual screening campaigns, thus rendering StreptomeDB a useful asset for the drug discovery community.

## Authors’ information

FNK is a computational chemist focused on natural product drug discovery and computer-aided drug design.

## Electronic supplementary material

Additional file 1: **Prepared 3D structures of compounds currently included in StreptomeDB with calculated “drug-likeness” descriptors.** (MDB 2 MB)

Additional file 2: **3D structures of the “drug-like” subset derived from the StreptomeDB library.** (MOL2 2 MB)

Additional file 3: **3D structures of the “lead-like” subset derived from the StreptomeDB library.** (MOL2 720 KB)

Additional file 4: **3D structures of the “fragment-like” subset derived from the StreptomeDB library.** (MOL2 208 KB)

Additional file 5: **3D structures of the total StreptomeDB dataset with calculated pharmacokinetic descriptors.** (SDF 13 MB)

## Abbreviations

3D: Three dimensional
ADME/T: Absorption, distribution, metabolism, elimination, and toxicity
CADD: Computer-aided drug design
DMPK: Drug metabolism and pharmacokinetics
log P: logarithm of the octan-1-ol/water partition coefficient
MDCK: Madin-Darby canine kidney
MW: Molar weight
NP: Natural product
NRB: Number of rotatable bonds
RBs: Rotatable bonds
VS: Virtual screening.
